# Root anatomical phenes predict root penetration ability and biomechanical properties in maize *(Zea Mays)*

**DOI:** 10.1093/jxb/erv121

**Published:** 2015-04-22

**Authors:** Joseph G. Chimungu, Kenneth W. Loades, Jonathan P. Lynch

**Affiliations:** ^1^Department of Plant Science, The Pennsylvania State University, University Park, PA 16802, USA; ^2^The James Hutton Institute, Invergowrie, Dundee, DD2 5DA, UK

**Keywords:** Anatomy, bending strength, phenes, tensile strength.

## Abstract

The ability of roots to penetrate hard soil is important for crop productivity but specific root phenes contributing to this ability are poorly understood. Root penetrability and biomechanical properties are likely to vary in the root system dependent on anatomical structure. No information is available to date on the influence of root anatomical phenes on root penetrability and biomechanics. Root penetration ability was evaluated using a wax layer system. Root tensile and bending strength were evaluated in plant roots grown in the greenhouse and in the field. Root anatomical phenes were found to be better predictors of root penetrability than root diameter per se and associated with smaller distal cortical region cell size. Smaller outer cortical region cells play an important role in stabilizing the root against ovalization and reducing the risk of local buckling and collapse during penetration, thereby increasing root penetration of hard layers. The use of stele diameter was found to be a better predictor of root tensile strength than root diameter. Cortical thickness, cortical cell count, cortical cell wall area and distal cortical cell size were stronger predictors of root bend strength than root diameter. Our results indicate that root anatomical phenes are important predictors for root penetrability of high-strength layers and root biomechanical properties.

## Highlight

Root bending, tensile strength, and ability to penetrate hard soil are related to anatomical phenes that are subject to selection in crop breeding programs.

## Introduction

Soil compaction adversely affects crop production in many parts of the world. The formation of strong or compacted soil layers restricts root exploration and access to nutrients and water thereby promoting early onset of stress (Barraclough and Weir, 1988). Soil strength and mechanical impedance to root growth typically increases with decreasing soil moisture (Cairns *et al.*, 2004; Iijima and Kato, 2007; Whitmore and Whalley, 2009). Impedance causes physical limitations to root growth in the rooting zone with a typical soil penetrometer resistance of 2.0MPa, the threshold for root elongation (Bengough *et al.*, 2011). Root growth in drying soil is generally constrained by a combination of mechanical impedance and water stress. However, soil strength increases nonlinearly with decreasing soil moisture, which may result in mechanical impedance limiting root growth to a relatively greater extent than water stress *per se* (Bengough *et al.*, 2011). Deep rooting is important for drought adaptation by improving utilization of water in deep soil strata (Lynch, 2013) but the presence of strong or compacted soil layers often precludes the exploitation of deep water and nutrients. Thus, deep roots that are capable of penetrating hard soil are important for improving crop productivity in compacted soils.

Soil management approaches to ameliorate subsoil compaction, such as deep ripping and the application of gypsum to improve soil structure and aggregate stability, are often used as a solution to soil compaction. However, in the long-term, these management practices may not be a good solution to alleviate soil compaction because they encourage decomposition of organic matter, breakdown of soil aggregates and weakening of soil structure (Brady and Weil, 2008) and are costly in terms of energy and time. The viable alternative is to find ways of developing root system traits capable of penetrating these hard soils and alleviating compaction with minimum cost, maintaining sustainability.

There is evidence of differences in root penetration ability between, and also within, species. Within-species variation in root penetration has been reported in cotton (May and Kasperbauer, 1999; Klueva *et al.*, 2000), maize and soybean (Bushamuka and Zobel, 1998), rice (Price *et al.*, 2000; Zheng *et al.*, 2000; Clark *et al.*, 2002), and wheat (Acuña and Wade, 2005; Botwright Acuna *et al.*, 2007; Kubo *et al*., 2006, 2008). Generally the ability of roots to penetrate hard soils is associated with phenes [‘phene’ is to ‘phenotype’ as ‘gene’ is to ‘genotype’ (Lynch, 2011)] that reduce the likelihood of root buckling when penetrating hard soils (Clark *et al.*, 2003; Bengough *et al.*, 2011; Jin *et al.*, 2013).

Mechanically, roots form an essential component in anchoring plants in the soil providing the foundations for plant stability, with most terrestrial plants dependent on their ability to stand upright. Root biomechanical properties have been of interest regarding their implications for the mechanics of plant anchorage and lodging resistance (Ennos *et al.*, 1993; Goodman and Ennos, 1998; Oladokun and Ennos, 2006). The resistance to lodging is a function of the resistance of the compressed leeward roots to bending, the anchorage of the windward roots under tension, the strength of the soil, and the mass of the soil-root plate (Coutts, 1986). Both attributes—tensile strength ‘the maximum force per unit area required to cause a material to break’ and bending strength—are thus key traits in enabling plants to resist lodging. In addition bending strength or stiffness is associated with good root penetration of hard soils (Clark *et al.*, 2008).

The maize root is comprised of the stele and cortex. The stele contains vascular bundles: the phloem and xylem. The cortex consists of a single layer of endodermis with casparian strips forming a barrier to the radial flow of water and nutrients with 8–15 layers of parenchymatous cortical cells. In this study the cortex is divided into three bands: the epidermis plus two or three layers beneath it forming the outer cortical region that can be thought of as the protective layer of the root; the middle 50% of the cortex or mesodermis; and the inner cortical region close to the endodermis.

Anatomical phenes have been associated with efficient soil resource acquisition under abiotic stresses; for example high root cortical aerenchyma (RCA), small living cortical area, reduced cortical cell file number, and large cortical cells (Fan *et al.*, 2003; Zhu *et al.*, 2010; Jaramillo *et al.*, 2013; Lynch, 2013; Lynch 2014; Chimungu *et al.*, 2014*a*,*b*; Lynch *et al.*, 2014, Chimungu *et al.*, 2015). The effects of root anatomical phenes on penetration and root biomechanics are not well understood (Lynch and Wojciechowski 2015). Some phenes such as RCA can overcome the effects of drought by improving soil exploration (Zhu *et al.*, 2010), but may also weaken the root structure (Engelaar *et al.*, 1993; Striker *et al.*, 2006). Reduced cortical cell file number (i.e. a thinner cortex) may reduce metabolic cost for soil exploration (Chimungu *et al.*, 2014*a*) but it may also entail signiﬁcant costs in terms of reduced mechanical strength. On the other hand, tissue made of smaller cells might have a higher tissue density providing rigidity and strength, and thus more resistance to buckling or rupture. However, no attempt has been made to relate variation in root anatomical phenes to root penetration ability and biomechanical properties. In this study we examine if biomechanical properties and anatomical phenes affect root penetrability of hard layers. We hypothesize that stele anatomical phenes influence root tensile strength, assuming that the cortical tissue is weaker compared to the stele; cortical traits influence root bending strength and root penetration, with penetrability driven by bending stiffness. The thicker cortex roots would presumably be less prone to buckling and develop the greater axial pressure necessary to penetrate the harder soil.

Given the potential for root anatomical phenes in improving crop adaptation to abiotic stresses, and the inconclusive nature of existing information on the effects of anatomical phenes on root biomechanical properties and penetrability, the objective of this study was to investigate the effects of anatomical phenes on root penetration ability of hard layers and biomechanical properties. Variation in anatomical phenes, root penetration ability, tensile strength and Young’s modulus of roots were quantified in growth chamber, greenhouse and field-grown plants. To our knowledge, this is the first mechanistic study on the relationship between root anatomical phenes and root penetrability of hard layers linked to biomechanical properties of maize.

## Materials and methods

### Experiment 1: evaluation of root penetration ability

Twenty-six maize genotypes contrasting in root anatomical traits were used to assess root penetration ability in a temperature-controlled growth chamber (Environmental Growth Chambers, Model GC-36, Chagrin Falls, OH44022, US) using a thin wax layer system (Taylor and Gardner, 1960; Yu *et al.*, 1995). Based on preliminary experiments, wax-petrolatum layers used in this study consisted of 60% wax (Royal Oak Sales, Inc, GA30076, US) and 40% petrolatum (Unilever, CT06611, US) by weight. The mixture was melted at 80°C, poured into molds and allowed to solidify at room temperature. The resulting wax-petrolatum disks were 125mm in diameter and 3mm thick (with equivalent strength of 1.7MPa at 27°C). Plants were grown in a randomized complete block design, with three replicates, in the temperature-controlled growth chamber. The mean minimum and maximum air temperatures during the experimental period were 25±3°C and 30±2°C, respectively with maximum illumination of 800 μmol photons m^-2^ s^-1^ and average relative humidity of 40%. Mesocosms were constructed of two stacked PVC pipes sealed together with duct tape, each 130mm long with an internal diameter of 10mm. The bottom pipe was capped, filled with 1279g of media (i.e. bulk density of 1.3g cm^-3^), and one wax-petrolatum layer placed on top with the top pipe placed onto the wax layer prior to filling with media. Growth medium consisted of (by volume) 50% commercial grade sand (Quikrete Companies Inc. Harrisburg, PA, USA), 25% vermiculite (Whittemore Companies Inc., Lawrence, MA, USA), and 25% topsoil (Hagerstown silt loam top soil, a fine, mixed, mesic Typic Hapludalf). Mineral nutrients were provided by mixing media with 10g per column of OSMOCOTE PLUS fertilizer (5–6 months release) (Scotts-Sierra Horticultural Products Company, Marysville, Ohio, USA) consisting of (%); NO_3_ (8) NH^+^_4_ (7), P (9), K (12), S (2.3), B (0.02) Cu (0.05), Fe (0.68), Mn (0.06), Mo (0.02), and Zn (0.05) for each column. Seeds were pre-germinated in a darkened germination chamber at 28±1°C, rolled in germination paper (Anchor Paper Company, St. Paul, MN, USA) and moistened with 0.5mM CaSO_4_ for 2 d. Two seedlings per mesocosm were transplanted, and thinned to one uniform seedling per mesocosm 3 d after planting. Planting was staggered by 1 d for each replicate. Top and bottom sections were hydraulically separated with the wax layer and moisture levels maintained by irrigating each layer separately. Plants were grown for 25 d and each replicate harvested in 1 d. Number of roots reaching the wax layer, numbers penetrating the wax layer, and total number of roots at the base of the stem were counted. Root penetrability was calculated as a ratio of the number of roots penetrating the wax-petrolatum layer to number of roots reaching the wax layer per plant; the ratio expressed as the root-penetration index (PR) (Materechera *et al.*, 1992). Three penetrated roots were sampled and preserved in 75% ethanol for quantification of anatomical traits.

### Experiment 2: evaluation of root tensile strength

Plants were grown in a greenhouse (February–March 2014) at University Park, PA, USA (40°4′N, 77°49′W), using 14/10h day/night: 23/20°C day/night: 40–70% relative humidity with natural light 500–1200 μmol photons m^-2^ s^-1^ PAR, and supplemental light 500–600 μmol photons m^-2^ s^-1^ PAR was provided with 400-W metal-halide bulbs (Energy Technics, York, PA, USA) for 14h/day. The mesocosms consisted of PVC cylinders 1.5 m in height by 0.15 m in diameter and lined with transparent hi-density polyethylene film to facilitate root sampling. The growth medium consisted of (by volume) 50% commercial grade sand (Quikrete Companies Inc. Harrisburg, PA, USA), 25% vermiculite and 25% topsoil [Hagerstown silt loam top soil (fine, mixed, mesic Typic Hapludalf)]. Mineral nutrients were provided by mixing the media with 70g per column of OSMOCOTE PLUS fertilizer (Scotts-Sierra Horticultural Products Company, Marysville, Ohio, USA) consisting of (%); N (15), P (9), K (12), S (2.3), B (0.02) Cu (0.05), Fe (0.68), Mn (0.06), Mo (0.02), and Zn (0.05) for each column. Seeds were germinated and planted as in Experiment 1 before thinning to one uniform seedling per mesocosm 5 d after planting.

Roots were sampled 40 d after planting with polyethylene liners extracted from mesocosms and laid on a root washing station. Root segments of 10cm were collected 0–20, 20–40, 40–60, 60–80, 80–100, 100–120, 120–140cm from the base of the primary, seminal, and first, second and third whorl crown roots (i.e. to assess the impact of root age on root tensile strength). Root segments were refrigerated at 4°C to preserve them for 24–30h until testing. Tensile measurements were carried out at the Mechanical Testing laboratory at The Pennsylvania State University using a universal testing machine (Instron, model 5866, Norwood, MA, USA). Prior to testing, all roots were inspected with damaged roots discarded. For tensile testing samples were clamped between two grips. Clamping is the most critical issue when measuring root strength. In our tests, the roots were clamped using wedges to avoid slippage and fine sandpaper was also attached to the clamps to increase friction. Each tested segment was 100mm in length allowing 50mm above and below to be clamped, minimizing potential slippage. Force was recorded during tensile testing with extension at a constant rate of 10mm min^-1^. Tensile load was measured using a 100 N load cell (Instron 2525–807 Series, Norwood, MA, USA) accurate to ±2.5 mN at maximum load. Root tensile strength was calculated as maximum tensile force, at ultimate failure, divided by root cross-sectional area for root tensile strength or by stele cross sectional area for stele tensile strength.

### Experiment 3: evaluation of root bending properties

Root bending properties of six field grown maize genotypes was quantified. Plants were grown at the Russell E Larson Agricultural Research Center in Rock Springs, PA, USA (40°42′N, 77°57′W,), during the summer of 2013. The soil is classified as a Hagerstown silt loam (fine, mixed, mesic Typic Hapludalf) with bulk density of 1.6g cm^-3^. Genotypes were grown in a randomized complete block design with three replications of each genotype. Each plot consisted of three rows, with each row being 2.5 m long, with 25cm spacing between plants and 75cm between rows. Three plants (i.e. flowering stage) were randomly excavated for analysis 60 d after planting. Six to eight root segments 10cm in length were collected 4cm from the base of each plant for bend tests. Before the tests all lateral roots were removed using a razor. Segments were placed between moist germination paper (Anchor Paper Co., St. Paul, MN, USA) and refrigerated at 4°C for 24–30h to preserve them until measurement, minimizing degradation. Three-point bending tests were carried out at The Pennsylvania State University Mechanical Testing laboratory, using a universal testing frame (Instron, model 5866, Norwood, MA, USA). Before testing, roots were inspected and damaged roots removed from the study. Samples were placed between two supports (set apart approximately 15 times the diameter of the sample) and a pushing probe of radius 10mm lowered until contact with the sample. During the test the crosshead was lowered at a rate of 10mm min^-1^, with peak bending force recorded. The force applied to the root was continuously registered by a 100 N (±2.5) load cell (Instron 2525–807 Series, Norwood, MA, USA).

### Root anatomical phene measurement

**Fig. 1.**
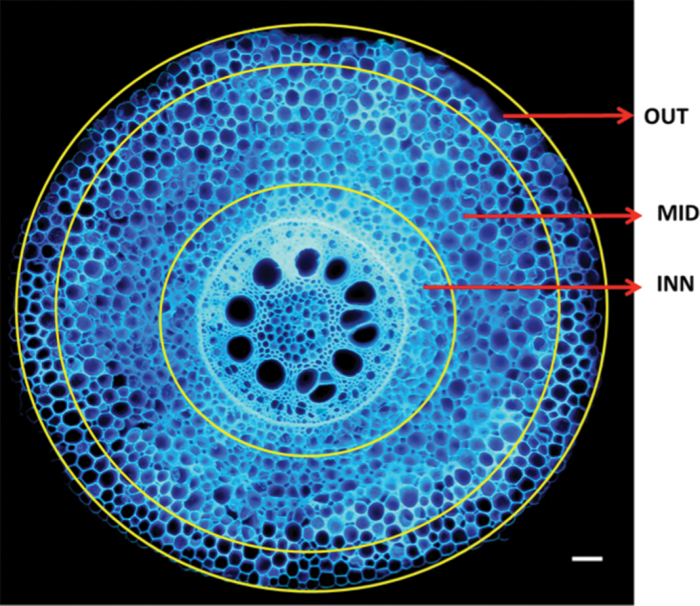
Maize root cross-section showing three cortical bands; outer cortex (OUT), middle cortex (MID), and inner cortex (INN). Bar=100 µm.

Root segments were ablated using laser ablation tomography (LAT) to obtain images for anatomical analysis. In brief, LAT is a semi-automated system that uses a laser beam (Avia 7000, 355nm pulsed laser) to vaporize or sublimate the root at the camera focal plane ahead of an imaging stage. The sample is incrementally extended into the beam, vaporized or sublimated, and imaged simultaneously. Imaging of root cross-sections was performed using a Canon T3i camera (Canon Inc. Tokyo, Japan) and 5× micro lens (MP-E 65mm) on the laser-illuminated surface. Image analysis was performed using *RootScan* software, an image analysis tool developed for analysing root anatomy (Burton *et al.*, 2012). Some of the primary anatomical phenes measured or calculated are presented in Table 1. For cell size determination cortex was divided into three bands (Fig. 1).

**Table 1.** Anatomical traits measured or derived using *RootScan* from laser ablated cross-section images

Trait by tissue region	Abbreviation
Root cross-section	
Root diameter (mm^2^)	RD
Cortex	
Total cortical area (mm^2^)	TCA
Cortical thickness (mm)	CT
Cortical cell wall area (mm^2^)	CCWA
Cortical cell file number	CCFN
Cortical cell count	CCC
Median cell size, inner cortex (μm^2^)	INN
Median cell size, middle cortex (μm^2^)	MID
Median cell size, outer cortex (μm^2^)	OUT
Root cortical aerenchyma (%)	RCA
Stele	
Stele diameter (mm)	SD
Stele cell wall area (mm^2^)	SCWA

### Data analysis

The R statistical package (R Development Core Team, 2014) was used for data analysis. Correlation analysis was used to test for relationships between root penetrability and bending strength and root anatomical phenes. To describe correlation patterns among phenes, principal components analyses (PCA) was performed for seven traits, using original data. This PCA helps elucidate the relationships among many traits simultaneously and summarizes them on a single graph. All traits that were significantly correlated to root penetration or bending strength were used as independent variables in a multiple linear regression to identify their contribution to root penetrability and bending strength; phenes with close interrelationships, or derived from each other, were excluded. The anova command was used to compare multiple regression models. Stepwise multiple linear regression procedure was used to identify phenes that correlated with root penetrability and bending strength variation. Final models were selected by using Akaike information criterion (AIC). Power law regressions were carried out to determine the relationship between root tensile strength and root diameter and stele diameter. The analysis of covariance (ANCOVA) was used to evaluate the effect of age on root tensile strength as influenced by diameter.

## Results

### Experiment 1. Relationship of root penetration and anatomical phenes in maize

**Fig. 2.**
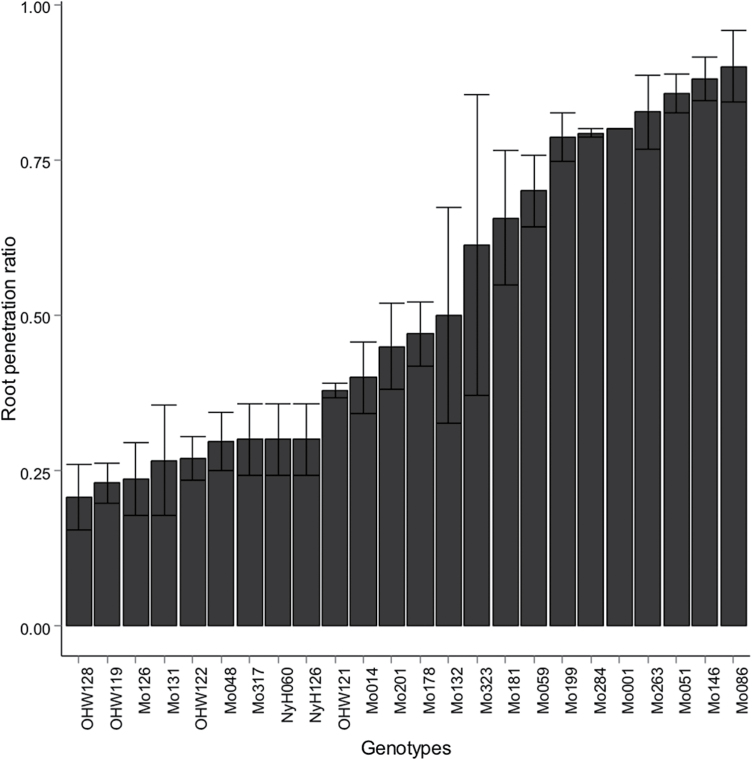
Root penetration (RP) of 26 maize genotypes ranked in ascending order. RP is calculated as the ratio of number of roots reaching the wax layer to number of roots that penetrated the wax layer, with an RP of 1 representing that all roots reaching the wax layer penetrated the layer.

Root penetration (RP) indicates the relative ability of a plant’s root to penetrate a wax layer. An RP value of 1 signifies that all roots penetrated the wax layer, while an RP value of 0 signifies that none of the roots that reached the wax layer were able to penetrate it. Across the 24 genotypes, there was 3-fold variation for RP, ranging from a minimum of 0.22 to a maximum of 0.90 (Fig. 2).

**Fig. 3.**
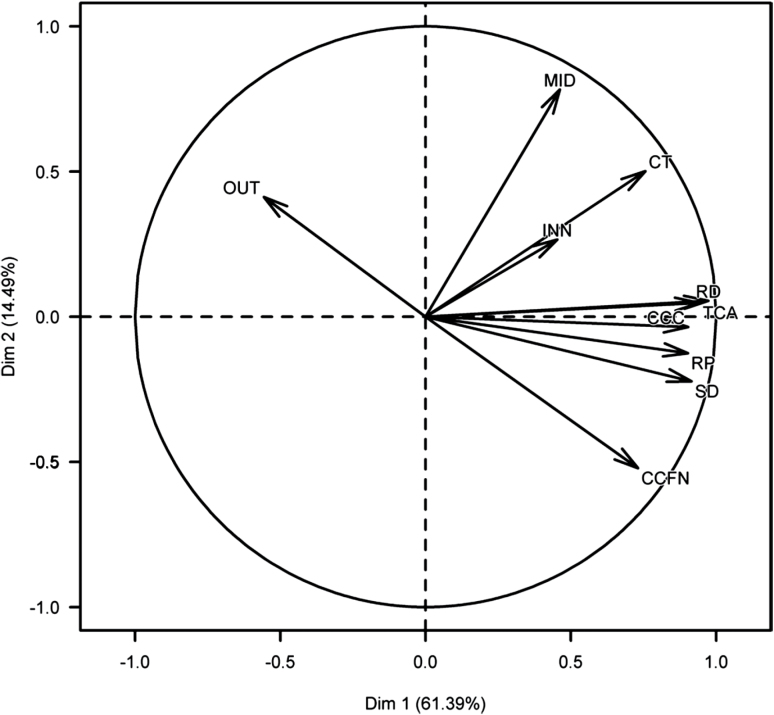
Principal component analyses (PCA) on genotype means for eight anatomical phenes among 24 genotypes. The angle between two arrows represents the correlation of the respective variables. There is no linear dependence if the angle is 90 degrees. RP, root penetration, RD, root diameter; SD, stele diameter; CT, cortex thickness, TCA, total cortical area; CCC, cortical cell count, INN, inner cortical cell size, MID, middle cortical cell size, OUT, outer cortical cell size, CCFN, cortical cell file number.

Multiple anatomical phenes were measured (Table 2) with substantial variation found in: total cortical area (TCA), cortex thickness (CT), cortical cell count (CCC), cortical cell file number (CCFN), cell size inner cortical region (INN), cell size mid-cortical region (MID), cell size in outer cortical region (OUT), and stele diameter (SD) (Table 2). Root diameter was correlated with root penetration (Supplementary Fig. S1). Several root phenes were strongly correlated with RD and RP (Fig. 3, Supplementary Table S1). Results of the PCA showed that the first axis explained 61.4% of the variation among the eight phenes and was mostly associated with TCA, CT, CCC, and SD (Fig. 3). The second axis explained 14.5% of the variation and was associated with CCFN, MID and OUT (Fig. 3). Specific Pearson’s correlations showed that both CT and SD were positively correlated with RD. Cortical phenes: TCA, CCC, CCFN, INN and MID were positively correlated with RD (Fig. 3, Supplementary Table S1), while DIS was negatively correlated with RD (r=−0.41, *P*<0.05) (Supplementary Table S1). The relationship between RD and RCA was not significant (Fig. 3, Supplementary Table S1). In addition, correlation analysis showed a significant and positive relationship between RP and SD, TCA, CCC, CCFN, CT, INN and MID (Fig. 3, Supplementary Table S1). Interestingly DIS was negatively correlated with RP (Supplementary Table S1). Among the anatomical phenes, some were negatively or positively correlated.

**Table 2.** Summary statistics with median, minimum (Min.), maximum (Max.) and fold-variation for root diameter (RD) and root anatomical phenes: total cortical area (TCA), cortex thickness (CT), cortical cell count (CCC), cortical cell file number (CCFN), cortical cell size in the outer cortical region (OUT), middle (MID) and inner cortical region of the cortex (INN) (see Fig. 1 for description), and stele diameter (SD) for 26 maize genotypes

Phene	Median	Min.	Max.	Fold-variation
RD	1.45	1	2.2	1.2
TCA	1.2	0.5	2.8	4.6
CT	0.4	0.3	0.6	1.0
CCC	770.5	273	2149	6.9
CCFN	11	7	15	1.1
INN	249.2	169	383.5	1.3
MID	408.55	300.8	786.5	1.6
OUT	238.5	102.8	487	3.7
SD	0.7	0.4	1.1	1.8

Root anatomical phenes significantly correlated with RP, i.e. TCA, CT, SD, CCC, TCA, CCFN and DIS were included in multiple regression analysis as independent variables. Three multiple regression models were used to explain variation in RP (Supplementary Table S2). The first model elucidated relationships between RP and RD plus anatomical phenes (Model 1), the second model was for RP and anatomical phenes (Model 2) and the third model was for RP and RD only (Model 3, Supplementary Fig. S1). Analysis of variance shows that removing RD from Model 1 (i.e. Model 2) does not significantly affect the fit of the model (*P*=0.78). Model 2 was significantly different from Model 3 (*P*<0.001). In addition Model 2 was a better model than Model 3 with a slightly greater coefficient of determination (0.79 *versus* 0.66). Anatomical phenes were a much better predictor for RP than root diameter *per se*. Stepwise multiple linear regression was applied to Model 2 to determine the anatomical phenes accounting for the majority of variability in RP. The lowest AIC stepwise model for RP included CT, OUT and SD, explaining 78% of the variability in PR (Table 3).

**Table 3.** Summary of multiple regression model (Model 2) of root penetration as predicted by cortex thickness (CT), cortical cell size of the outer cortical region (OUT) and stele diameter (SD); SE is the standard error of the coefficients, *P≤0.05; ***P≤0.001

	Coefficient	SE	
(Intercept)	−0.2543	0.1196	*
CT	0.8779	0.2220	***
OUT	−0.0007	0.0002	***
SD	1.1170	0.1595	***
R^2^	0.788		
Adjusted R^2^	0.776		

### Experiment 2. Relationship of root tensile strength and anatomical phenes in maize

**Fig. 4.**
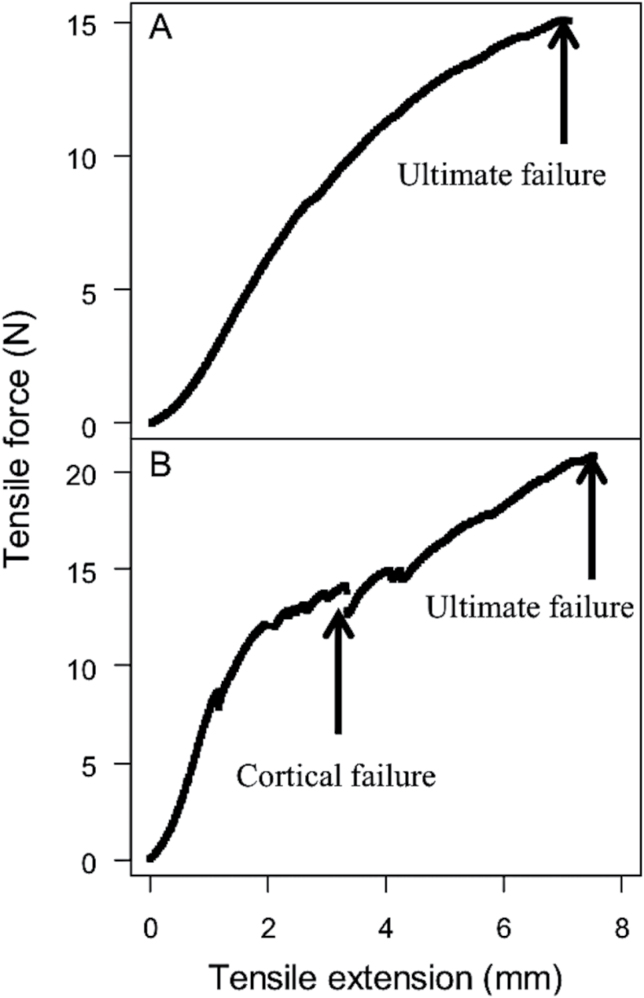
Force-displacement curves (A) with a single peak representing root ultimate failure, and (B) with multiple peaks, the first peak value is due to failure of the cortex and the second peak is the ultimate root failure.

**Fig. 5.**
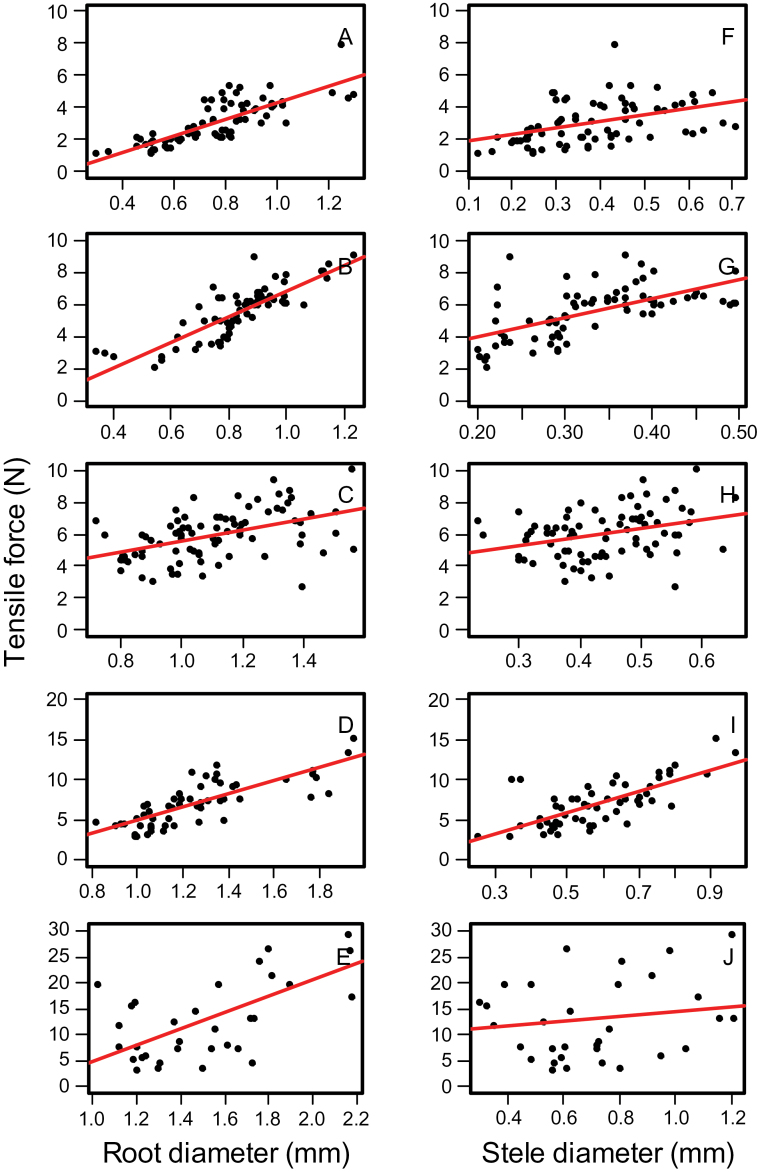
The relationship between tensile force (N) and root diameter (A–E) and stele diameter (F–J) for different root types: primary (A and F), seminal (B and G), first crown root (C and H), second crown root (D and I), and third crown root (E and J), 45 d after planting. Solid lines are fitted linear regression lines. See Table 5 for regression coefficients and significance levels.

**Fig. 6.**
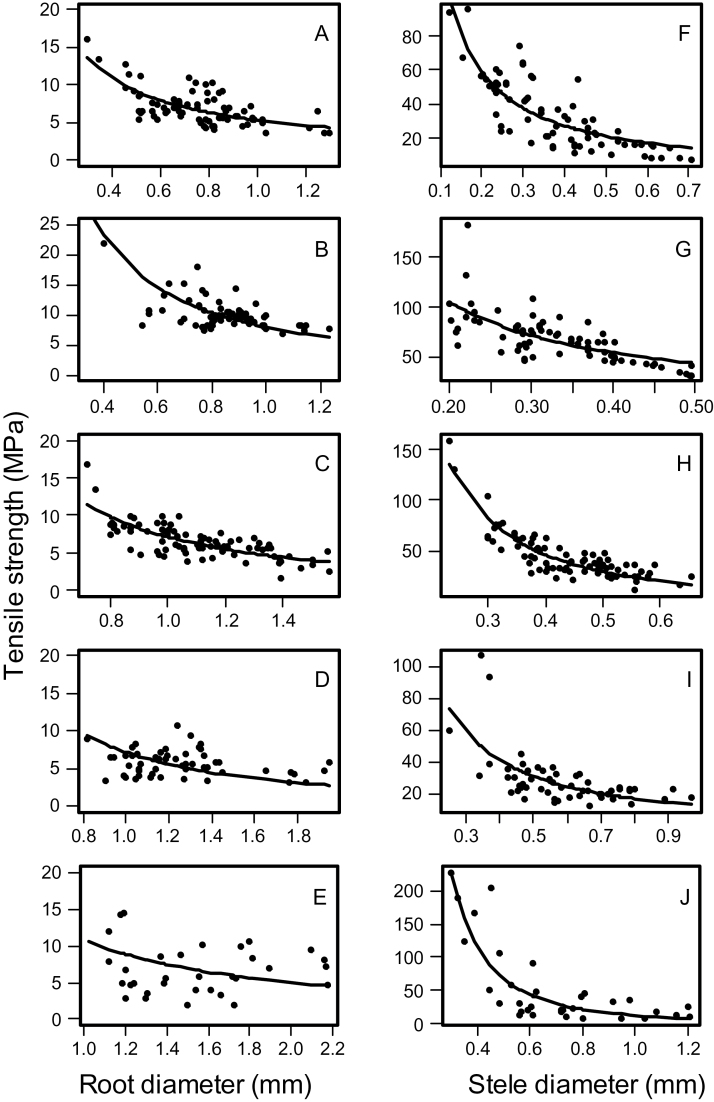
The relationship between tensile strength and root diameter (A–E) and stele diameter (F–J) for different root types: primary (A and F), seminal (B and G), first crown root (C and H), second crown root (D and I), and third crown root (E and J), 45 d after planting. Solid lines are fitted curvilinear negative power regression lines. See Table 4 for regression coefficients and significance levels.

The root diameter of the tested samples ranged from 0.67 to 1.92mm (Supplementary Table S3). During tensile testing roots displayed typical elastic-plastic deformation with initially steep stress-strain curves in the elastic region before plastic deformation beyond the yield point. At the final stages of the test, the irregular sounds of the cortex snapping were heard in some samples prior to ultimate tensile failure. Two distinct peak values were observed in the force-displacement curves (Fig. 4). The first peak value is due to failure of the cortex with the second peak ultimate root failure (i.e. failure of the stele in tension) (Fig. 4). The results showed that the tensile breaking force (TBF) increased with increasing root and stele diameter (Fig. 5). Accordingly, tensile strength was calculated based on root cross-sectional area (root tensile strength) and stele cross-sectional area assuming that tensile force was concentrated within the stele only (stele tensile strength) with no load on the cortex. Relationships between the tensile strength and either root diameter or stele diameter were fitted with power-law relationships (Fig. 6). Table 4 presents the parameters and the coefficient of determination for the fitted models (tensile strength = *ad* ^*b*^ where *d* is diameter, *a* and *b* are regression coefficients). In general increasing stele or root diameter were both associated with decreased tensile strength (Fig. 6) following a power law equation for all root classes. Stele diameter was a stronger predictor of tensile strength than root diameter with a greater coefficient of determination (Fig. 6, Table 4).

**Table 4.** Parameters (*a* and *b*) and coefficient of determination (*R* ^2^) values for the power regression, expressing the decrease in root tensile strength and stele tensile strength with increasing root diameter and stele diameter for different root types in maize; *n* indicates number of samples

Root tensile strength				
Root type	*a*	*b*	*n*	*R* ^2^
Primary	5.33	−0.77	71	0.39
Seminal	8.02	−1.16	70	0.52
Nodal1	7.11	−1.42	77	0.51
Nodal2	6.01	−0.29	54	0.06
Nodal3	10.86	−1.11	32	0.08
Stele tensile strength				
Primary	9.68	−1.12	71	0.58
Seminal	22.47	−0.95	70	0.59
Nodal1	7.31	−2.01	77	0.55
Nodal2	12.97	−1.26	54	0.42
Nodal3	12.71	−2.41	32	0.52

Root tensile strength decreased with increasing distance from the stem base (younger tissue) in all root types (Table 5). ANCOVA showed that tensile strength was signiﬁcantly affected by age (i.e. distance from the stem base) with regard to root diameter in seminal, second and third order nodal roots, while the relationship was not significant in primary and first nodal roots (Table 5).

**Table 5.** Summary of analysis of covariance models (F-value and degrees of freedom) of root tensile strength as influenced by age (distance from the stem base), root diameter and stele diameter; *P≤0.05; **P≤0.01; ***P≤0.001

	Tensile strength-Root diameter				
	Primary	Seminal	Nodal1	Nodal2	Nodal3
Age	1.1(1,72)	9.1(1,69)**	0.006(1,76)	5.3(1,51)*	6.6(1,29)*
Root diameter	23.6(1,72)***	0.3(1,69)	18.2(1,76)***	20.7(1,51)***	4.3(1,29)*
Age*Root diameter	0.4(1,72)	10.8(1,69)**	1.8(1,76)	1.2(1,51)	3.1(1,29)
*R* ^2^	0.41	0.53	0.67	0.36	0.43
	Tensile strength-Stele diameter				
Age	7.5(1,72)**	8.1(1,69)**	5.8(1,76)*	1.5(1,51)	26.6(1,29)***
Root diameter	43.1(1,72)***	43.5(1,69)***	31.2(1,76)***	24.1(1,51)***	24.3(1,29)***
Age*Root diameter	0.5(1,73)	2.3(1,69)	0.9(1,76)	0.3(1,51)	12.1(1,29)**
*R* ^2^	0.74	0.73	0.68	0.63	0.43

### Experiment 3. Root bending strength is related to cortical anatomy

**Fig. 7.**
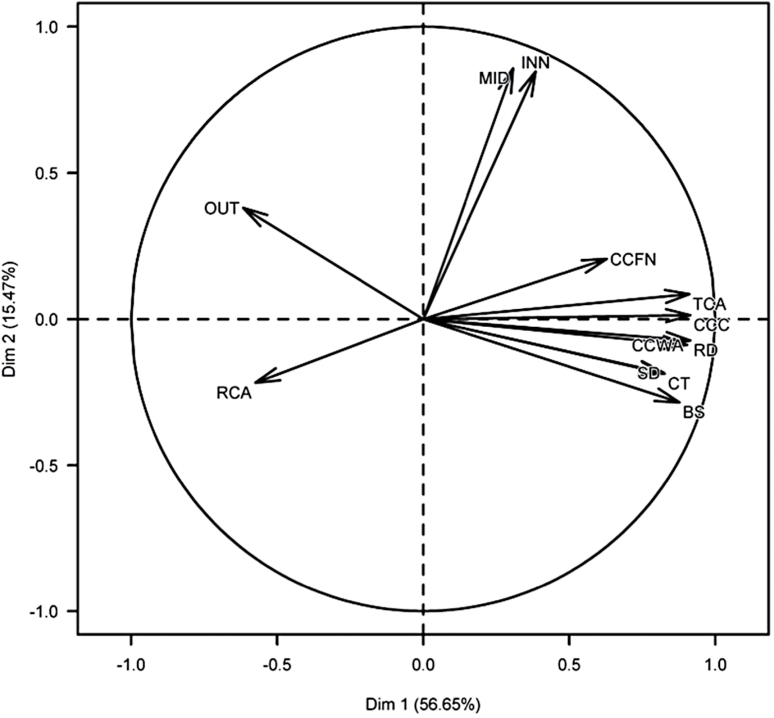
Principal component analyses (PCA) for eight anatomical phenes. The angle between two arrows represents the correlation of the respective variables. There is no linear dependence if the angle is 90 degrees. BS, root bending strength, RD, root diameter; SD, stele diameter; CT, cortex thickness, TCA, total cortical area; RCA, root cortical aerenchyma, CCC, cortical cell count, INN, inner cortical cell size, MID, middle cortical cell size, OUT, outer cortical cell size, CCFN, cortical cell file number.

Root bending strength was positively correlated with RD, CCWA, CT, SD, TCA, CCC, and CCFN whereas it was negatively correlated with RCA and OUT (Supplementary Table S4, Supplementary Fig. S2). No significant correlations were found between bending strength and either INN or MID (Supplementary Table S4). PCA showed that the first axis explained 56.6% of the variation among the eight phenes and was mostly associated with TCA, CT, CCC, CCWA and SD (Fig. 7). The second axis explained 15.4% of the variation and was associated with INN and MID (Fig. 7).

Root anatomical phenes significantly correlated with bending strength were included in multiple regression analysis to determine which were most important in predicting bending strength; with CCWA, CT, SD, CCC, TCA, CCFN, RCA and OUT as independent variables. Three multiple regression models were used to explain variation in bending strength (Supplementary Table S5). Model 1 assumes that bending strength can be accurately predicted by RD plus anatomical phenes: Model 1 accounted for 77% of the variation. Model 2 assumes that bending strength can be accurately predicted by root anatomical phenes alone: Model 2 accounted for 71% of the variation. Model 3 assumes that bending strength can be accurately predicted by RD alone: this model accounting for 47% of the variation (Supplementary Fig. S2). Results suggest that anatomical phenes were better predictors of bending strength than RD alone. The parsimonious model, based on Model 2, for root bending strength included CCC, OUT, CT and CCWA, explaining 86% bending strength variation (Table 6).

**Table 6.** Summary of multiple regression model of root bending strength as predicted by cortical cell wall area (CCWA), cortical cell count (CCC), cortex thickness (CT), and cortical cell size in the outer cortical region (OUT); *P*≤0.05

	Coefficient	Std. Error	Pr(>|t|)
(Intercept)	0.88	19.40	0.964
CCWA	7.19	3.22	0.0324 *
CCC	0.01	0.00	0.0106 *
CT	53.88	20.63	0.0134 *
OUT	−0.30	0.12	0.0130 *
R^2^	0.86		
Adjusted R^2^	0.84		

## Discussion

Root diameter is considered an important trait that strongly influences root biomechanical properties and the ability of roots to penetrate hard soil. We demonstrated that root anatomical phenes are better predictors of RP, tensile strength and bending strength than root diameter *per se*. Root tensile strength decreases with increasing diameter according to a negative power law (Bischetti *et al.*, 2005; Loades *et al.*, 2013), thicker roots are associated with good penetration of strong soils by relieving stress at the growing root tip (Hettiaratchi, 1990; Kirby and Bengough, 2002). Additionally, thicker roots are more resistant to buckling and deflection when encountering hard soil domains (Whiteley *et al.*, 1982; Clark *et al.*, 2003; Jin *et al.*, 2013). Clark *et al.* (2008) reported log-log positive relationships between root diameter and bending stiffness.

RP was evaluated using wax-petrolatum layers, which simulate strong soil. The use of a wax layer system proved to be a suitable method for detailed root penetration evaluation in maize. The advantage of the wax-layer system is that physical properties of wax are not affected by changes in moisture content that alter the strength of artificially compacted soil (Yu *et al.*, 1995). This type of system has been successfully used in the identification of rice genotypes capable of penetrating strong soils (Yu *et al.*, 1995; Clark *et al.*, 2000, 2002; Zheng *et al.*, 2000), and wheat (Acuña and Wade, 2005; Botwright Acuna *et al.*, 2007), with results on rice confirmed in field trials (Samson *et al.*, 2002).

A particularly interesting observation in this study was the strong negative relationship between RP and OUT. The presence of smaller cells in this region might play an important role in stabilizing the root against ovalization or compression and thereby reducing the risk of local buckling and collapse during penetration. Root resistance to compression is known to increase with the thickness of the multiseriate epidermal layer (Striker *et al.*, 2007). We observed that the smaller cells in the outer cortical region have thick cell walls and are closely packed, which may make the root stronger and more resistant to compression deformation due to external forces.

Integrating the preceding concepts and taking rhizoeconomics and the distribution of soil resources into account, the ‘steep, cheap and deep’ ideotype has been useful for identifying phenes for improving resource acquisition under edaphic stress (Lynch, 2013; 2014). Many elements of this ideotype are also relevant for improving root penetration ability in hard soils, such as steep growth angles, which can improve root penetration by deploying roots near vertical incidences with compacted soil layers, thereby reducing the probability of buckling, and large diameter, which may also improve root penetration as discussed below (Lynch and Wojciechowski 2015). However, the structural investments and metabolic costs of root systems are substantial and can exceed half of daily photosynthesis (Lambers *et al.*, 2002). Root anatomical phenes influence the metabolic costs of soil exploration (Zhu *et al.*, 2010; Jaramillo *et al.*, 2013; Lynch 2014; Lynch *et al.*, 2014). These phenes include root cortical aerenchyma (RCA), living cortical area (LCA), cortical cell file number, and cortical cells. We suggest that an anatomical root ideotype for greater root penetration in hard soils should include (i) small outer cortical region cells to provide mechanical reinforcement for the root to resist bending or buckling when penetrating hard layers, (ii) large cortical cells in the mesodermis to reduce the metabolic cost of soil exploration (Lynch, 2013; Chimungu *et al.*, 2014*b*); (iii) thick axial roots with more aerenchyma to reduce root metabolic cost, in favour of root growth to penetrate hard soils. The presence of aerenchyma will not affect root penetration ability since RCA forms in mature root tissue behind the zone of active root elongation and root hair formation. It is important to note that studies have demonstrated that the penetration of roots through soils with a large amount of fine particles (silt and clay) and densely compacted soils is probably achieved through the possession of large root diameter, which resists buckling, while in coarse-textured sandy or well-structured soils thin roots would penetrate the soil more easily through gaps between soil aggregates and large pores (Scholefied and Hall, 1985; Pietola and Smucker, 1998; Allah *et al.*, 2010).

The utility of phenes is affected by the external environment as well as the plant phenotype in which it is expressed. Knowledge of interactions among phenes is essential in developing an ideotype for optimizing root penetration. These phene interactions can be additive, synergistic or antagonistic (York *et al.*, 2013). For example, root hairs have been linked to good root penetration of hard soils by providing anchorage to the growing root tip (Haling *et al.*, 2013), and steep root angles are also associated with improved root penetration (Dexter and Hewitt, 1978; Whalley *et al.*, 2012). We suggest synergisms or additive interactions between root phenes such as root growth angle, root hairs and anatomical phenes, because a combination of greater anchorage of the root tip, reduced axial stress, and resistance to bending or buckling would work together to improve root penetrability of hard soils.

Root tensile strength is another key factor in understanding and predicting plant anchorage and contributions to soil stabilization. Root strength decreases with increasing root diameter; one explanation may be cellulose content in small diameter roots (Genet *et al.*, 2005), or it may be due to autocorrelation with root diameter as tensile stress is calculated using diameter (Hales *et al.*, 2009). Abiotic stress has also been found to influence root biomechanical properties (Loades *et al.*, 2013) and it is possible that soil physical heterogeneity may also account for differing biomechanical properties.

Karrenberg *et al.* (2003) argued that other root parameters may be better predictors of tensile strength than root diameter, especially in roots with a thick cortex. The potential implications of cortical failure during tensile testing reduces the area over which an applied force acts, so the actual tensile stress will be greater than that calculated based on root diameter. Indeed, calculated tensile stress based on stele area was generally greater than calculated stress based on root cross-sectional area, as would be expected due to radial diameter reduction. The strength of the stele may be attributable to greater cellulose content, which forms long chain polymers in the cell walls of root xylem tissue (Genet *et al.*, 2005). Although lateral roots were removed from root samples prior to testing they can anchor the cortex to the stele. It is of interest to evaluate the effect of lateral root density on individual root tensile strength and this merits further research.

Root biomechanical properties change with root age (Genet *et al.*, 2005; Loades *et al.*, 2013). In this study we found that root tensile strength decreased with increasing distance from the stem base, showing an age effect (Table 5). Maize, like most monocots, lacks secondary root growth. The change in tensile strength with distance from the stem base is likely attributable to age or root development. These results are consistent with previous studies in other grasses, with strength decreasing with distance from the base of the stem (Ennos *et al.*, 1993; Easson *et al.*, 1995; Loades *et al.*, 2013).

Bending tests revealed potentially interesting variations in terms of root stiffness. By comparing stress/strain curves beyond the yield point, curves were interrupted by one or more small steps indicating localized failures and probable fractures of the cortex. Previous studies showed that bending strength is associated with root diameter (Clark *et al.*, 2008). In this study cortical traits were better predictors for root bending strength than root diameter *per se*. This is consistent with our observations that the cortex failed first during bending test (Supplementary Fig. S3). Since mechanical stresses are additive, the failure of the peripheral tissue (i.e. the cortex) is expected to decrease root stiffness, so the stele has a minor role in bending strength. The inclusion of DIS in the stepwise model for bending strength is consistent with previous studies that have shown that, in the mature maize root system, roots are strengthened near the base by a heavy lignified exodermis, which makes them rigid in bending (Ennos *et al.*, 1993; Striker *et al.*, 2007). These results provide further evidence that in order to fully understand root biomechanical properties it is necessary to consider the various functional phenes simultaneously and attempt to unravel causal relationships among them.

Root biomechanical properties are influenced by a number of different factors and also by the soil environment. Soil physical conditions such as mechanical impedance affect biomechanical properties of roots. Goodman and Ennos (1999) found that bending strength of maize roots changes with soil bulk density, with roots from stronger soil being less stiff than those from weak soil. In addition Pollen and Simon (2005) reported that change in soil moisture content, soil texture and nutrient status, can affect root tensile strength. Considering those effects, caution must be taken when interpreting such relationships like those reported in this study without taking into consideration the growth conditions. In this study plants were grown in the field and in the greenhouse under high light intensity using uniformly packed mesocosms. Topsoil was added to the growth medium in the greenhouse to mimic soil conditions in the field. We therefore propose that our experimental conditions are not likely to be artifactual.

## Conclusion

Root diameter is a function of both stele diameter and cortical thickness. We found that cortical thickness is important for bending, while stele diameter is important for tensile strength. However, many other anatomical phenes were stronger predictors of root penetration and biomechanical properties than root diameter. More work is required to fully understand the effect of root anatomical phenes on root penetrability in field. Researchers of these properties should consider root anatomy. Because anatomical phenes are more elemental than aggregate traits like root diameter they are more likely to be under simpler genetic control, and may be more fruitful selection criteria in crop breeding programmes.

## Supplementary data

Supplementary data can be found at *JXB* online.

Supplementary Fig. S1. Correlation between root diameter (mm) and penetration for roots of maize genotypes grown in temperature-controlled growth chamber.

Supplementary Fig. S2. Correlation between root diameter (mm) and bending strength (Nm) for roots of maize genotypes grown in field

Supplementary Fig. S3. Cross-section of root segment following tensilometry.

Supplementary Table S1. Correlation coefficients for root penetration and anatomical traits of 24 maize genotypes

Supplementary Table S2. Summary of a multiple regression models of root penetration ability as predicted by root anatomical phenes and diameter.

Supplementary Table S3. Root diameter with distance from the stem base for different root types: primary, seminal, first crown root (Nodal1), second crown root (Nodal2) and third crown root (Nodal3).

Supplementary Table S4. Correlation coefficients between root bending strength and anatomical phenes.

Supplementary Table S5. Summary of a multiple regression models of root bending strength as predicted by root anatomical phenes and diameter.

Supplementary Data
