# Supplementary material for: Root anatomical phenes predict root penetration ability and biomechanical properties in maize (Zea Mays)
Source: J Exp Bot. 2015 Apr 22;66(11):3151–62. doi: 10.1093/jxb/erv121 (PMC4449537; doi:10.1093/jxb/erv121)
Supplement: Supplementary Data [file supp_66_11_3151__index.html]

Root anatomical phenes predict root penetration ability and biomechanical properties in maize (Zea Mays) — Root anatomical phenes predict root penetration ability and biomechanical properties in maize (Zea Mays) — Supplementary Data 

# Root anatomical phenes predict root penetration ability and biomechanical properties in maize *(Zea Mays)*

## Supplementary Data

Data files

**Files in this Data Supplement:**

- Supplementary Data - Supplementary Data
