# Supplementary material for: Root anatomical phenes predict root penetration ability and biomechanical properties in maize (Zea Mays)
Source: J Exp Bot. 2015 Apr 22;66(11):3151–62. doi: 10.1093/jxb/erv121 (PMC4449537; doi:10.1093/jxb/erv121)
Supplement: Supplementary Data [file supp_erv121_jexbot138693_file001.pdf]

Title: Root anatomical phenes predict root penetration ability and biomechanical properties in maize

Authors: Joseph G Chimungu, Kenneth W Loades, and Jonathan Lynch

Supplemental Tables

Supplemental table S1. Correlation coefficients for root penetration and anatomical traits of 24 maize genotypes. PR: root penetration; SD: Stele diameter; CCFN: cortical cell file number; CT: cortical thickness; OUT: cell size of the outer cortical region; MID: cell size of the middle 50% of the cortex; INN: cell size of the inner cortical region; CCC: number cortical cells. RCA: Root cortical aerenchyma

	RD	TCA	CT	RCA	CCC	CCFN	INN	MID	OUT	SD
RD	1									
TCA	0.96**	1								
CT	0.88**	0.93**	1							
RCA	-0.18ns	-0.19ns	-0.18ns	1						
CCC	0.87**	0.87**	0.71**	-0.28*	1					
CCFN	0.64**	0.56**	0.36*	-0.06ns	0.69**	1				
INN	0.24*	0.2*	0.21*	0.06ns	0.06ns	0.03ns	1			
MID	0.39*	0.34*	0.55	-0.3*	0.33*	0.01ns	0.29	1		
OUT	-0.41**	-0.41**	-0.23*	0.08ns	-0.3*	-0.35**	-0.23	0.13ns	1	
SD	0.85**	0.77**	0.51**	-0.11ns	0.82**	0.77**	0.21	0.25*	-0.4**	1
PR	0.82**	0.75**	0.6**	-0.13ns	0.68**	0.62**	0.3*	0.28*	-0.57**	0.82***

. *Significant at P < 0.05, **Significant at P < 0.01.

Supplemental table S2. Summary of a multiple regression models of root penetration ability as predicted by root anatomical phenes and diameter.

Model 1<-lm(RP~RD+TCA+CT+CCC+CCFN+INN+MID+OUT+SD)

	Estimate	Std. Error
(Intercept)	-3.53E-01	2.59E-01
RD	3.07E-01	1.04E+00
TCA	-3.24E-02	2.41E-01
CT	2.36E-01	1.20E+00
CCC	-1.18E-04	9.26E-05
CCFN	7.66E-03	1.18E-02
INN	1.24E-04	2.69E-04
MID	1.91E-04	1.52E-04
OUT	-7.35E-04	1.98E-04
SD	7.35E-01	8.08E-01
Adjusted R^2	0.708	

Model 2<-lm(RP~TCA+CT+CCC+CCFN+INN+MID+OUT+SD)

	Estimate	Std. Error
(Intercept)	-2.91E-01	1.52E-01
TCA	3.66E-02	5.93E-02
CT	5.76E-01	3.32E-01
CCC	-1.31E-04	8.11E-05
CCFN	8.61E-03	1.13E-02
INN	1.28E-04	2.67E-04
MID	1.89E-04	1.50E-04
OUT	-7.24E-04	1.93E-04
SD	9.66E-01	2.05E-01
Adjusted R^2	0.650	

Model 3<-lm(RP~RD)

	Estimate	Std. Error
(Intercept)	-0.40	0.07
RD	0.65	0.05
Adjusted R^2	0.462	

Supplemental table S3. Root diameter with distance from the stem base for different root types: primary, seminal, first crown root (Nodal1), second crown root (Nodal2) and third crown root (Nodal3).

	Root diameter (mm)

Distance from the stem base	Primary	Seminal	Nodal1	Nodal2	Nodal3
20	0.93±0.29a	0.95±0.15a	1.16±0.24a	1.67±0.23a	1.92±0.27a
40	0.68±0.13b	0.85±0.08ab	1.15±0.11a	1.27±0.18b	1.33±0.28b
60	0.77±0.09ab	0.76±0.09bc	1.12±0.19a	1.21±0.1bc	1.37±0.22b
80	0.79±0.18ab	0.67±0.19c	1.09±0.21a	1.12±0.12bc	1.45±0.21b
100	0.67±0.14b	0.85±0.2ab	0.99±0.18a	1.1±0.14bc	1.43±0.23b
120	0.68±0.15b		0.82±0.043a	0.99±0.07c	
		Tensile strength –calculated based on root diameter (MPa)			
20	9.13±2.8a	25.55±6.9a	9.44±2.2a	7.786±1.6a	13.74±5.7a
40	8.12±3.1ab	10.69±2.1b	6.38±1.3b	6.2±1.4ab	10.53±3.2ab
60	7.68±1.2ab	9.854±1.6b	5.83±1.7b	6.01±1.2abc	5.82±3.4b
80	6.69±1.5abc	8.762±0.6b	5.56±1.3b	5.4±1.7abc	5.32±2.2b
100	5.42±0.5bc	8.309±1.2b	5.73±0.9b	5.254±0.3bc	4.65±1.3b
120	5.38±1.6c		4.42±1.5b	4.267±0.9c	
		Tensile strength –calculated based on stele diameter (MPa)			
20	55.27±21.4a	92.75±62.1a	55.27±21.4a	25.9±4.4a	147.9±56.6a
40	48.04±19.1a	79.97±20.2a	48.04±19.1a	28.08±9.4a	140±84.6a
60	41.42±17.6ab	73.53±13.4a	41.42±17.6ab	27±6.2a	44.14±44.2b
80	28.98±11.8bc	69.01±12.1a	28.98±11.8bc20	35.9±29.2a	21.42±5.9b
100	31.76±12.3abc	40.85±8.1b	31.76±12.3abc40	21.98±9.3a	16.23±5.1b
120	18.36±11.4c		18.36±11.4c60	25.49±12.9a	

Supplemental table S4. Correlation coefficients between root bending strength and anatomical phenes. BS: root bending strength, CCFN: cortical cell file number, OUT: cell size of the outer cortical region, MID: cell size of the middle 50% of the cortex, INN: cell size of the inner cortical region, CCA: Cortical cell area, CCC: number cortical cells, RCA: Root cortical aerenchyma, SD: Stele diameter, CT: cortical thickness, CCWA: Cortical cell wall area

	RD	CCWA	TCA	CT	SD	RCA	CCC	INN	MID	OUT	CCFN	BS
RD	1											
CCWA	0.67**	1.00										
TCA	0.84**	0.68**	1.00									
CT	0.39**	0.57**	0.42**	1.00								
SD	0.75**	0.52**	0.68**	0.34**	1.00							
RCA	-0.19ns	-0.19*	-0.30*	-0.07ns	-0.29*	1.00						
CCC	0.61**	0.55**	0.58**	0.38**	0.52**	-0.50**	1.00					
INN	0.16ns	0.15ns	0.24*	0.13*	0.07ns	-0.22*	0.20*	1.00				
MID	0.09ns	0.06ns	0.18*	0.07ns	0.01ns	-0.15ns	0.14ns	0.55**	1.00			
OUT	-0.30**	-0.37**	-0.31*	-0.52*	-0.29*	0.25**	-0.36**	0.00ns	0.10**	1.00		
CCFN	0.43**	0.42**	0.43**	0.38**	0.32**	-0.40**	0.57**	0.39**	0.26*	-0.29*	1.00	
BS	0.51**	0.67**	0.47**	0.66**	0.42**	-0.23*	0.54**	0.07ns	-0.02ns	-0.53**	0.36**	1.00

. *Significant at P < 0.05, **Significant at P < 0.01.

Supplemental table S5. Summary of a multiple regression models of root bending strength as predicted by root anatomical phenes and diameter.

Model 1<-lm(BS~RD+TCA+CT+CCC+CCFN+RCA+INN+MID+OUT+SD)

	Estimate	Std. Error
(Intercept)	-0.82	1.57
RD	1.54	0.56
TCA	-0.53	0.18
CT	4.04	1.12
CCC	0.0002	0.0002
CCFN	-0.05	0.03
RCA	4.57	8.53
INN	0.007	0.004
MID	-0.001	0.002
OUT	-0.01	0.007
SD	-0.08	1.00

Model 2<-lm(BS~TCA+CT+CCC+CCFN+RCA+INN+MID+OUT+SD)

	Estimate	Std. Error
(Intercept)	1.68	1.40
TCA	-0.17	0.14
CT	3.67	1.224
CCC	0.0004	0.0002
CCFN	-0.05	0.04
RCA	5.86	9.34
INN	0.003	0.004
MID	-0.007	0.002
OUT	-0.01	0.008
SD	1.22	0.97

Model 3<-lm(BS~RD)

	Estimate	Std. Error
(Intercept)	-0.14	0.04
RD	0.96	0.15

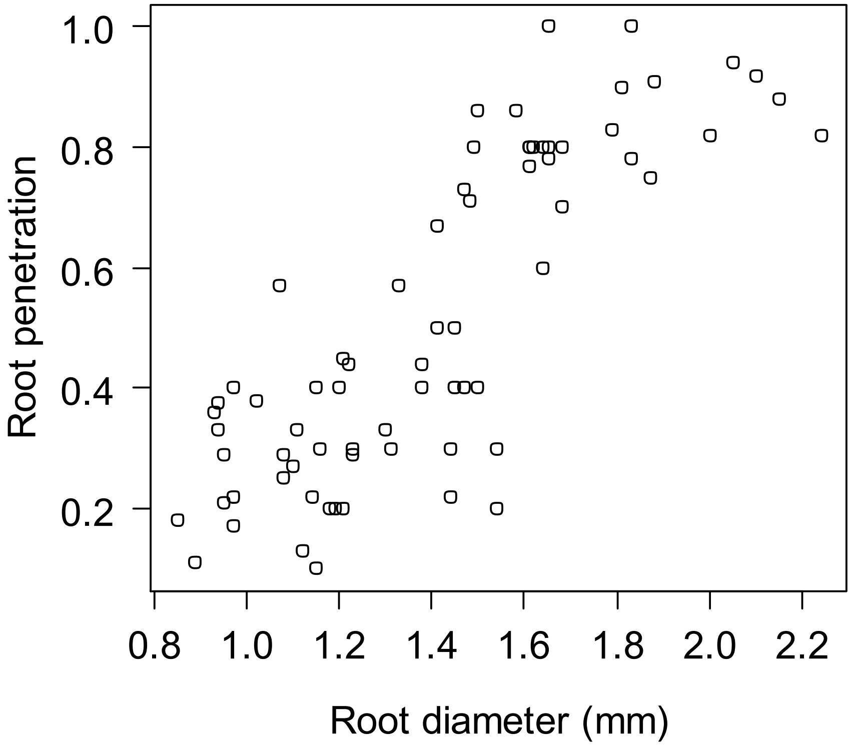

Supplemental figure 1. Correlation between root diameter (mm) and penetration for roots of maize genotypes grown in temperature-controlled growth chamber. Each point represents a single plant.

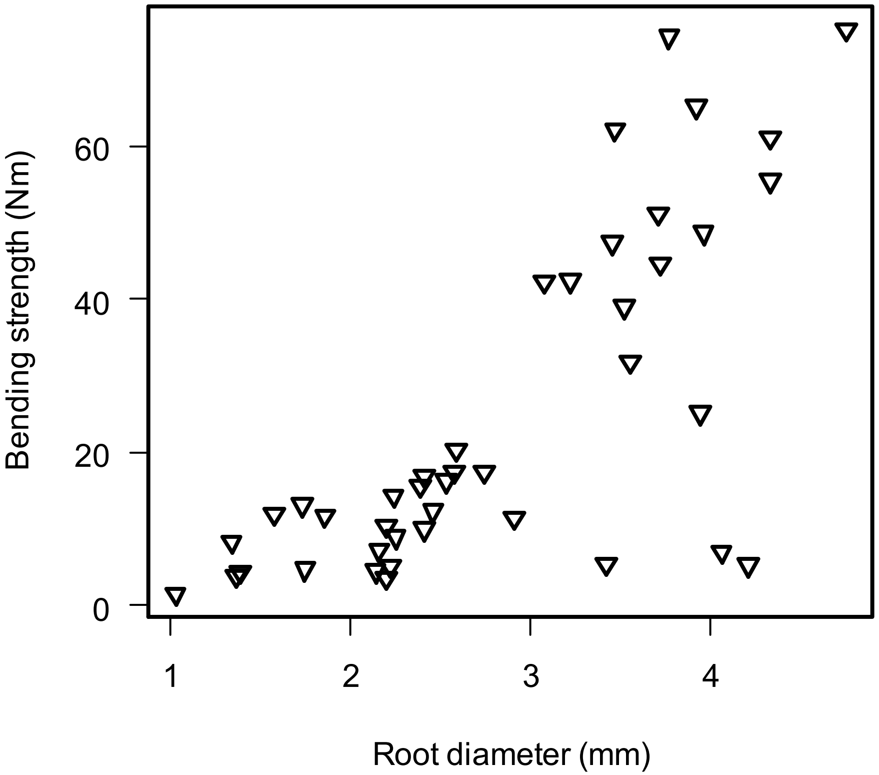

Supplemental figure 2. Correlation between root diameter (mm) and bending strength (Nm) for roots of maize genotypes grown in field and samples were collected 70 days after planting. Each point represents a single root.

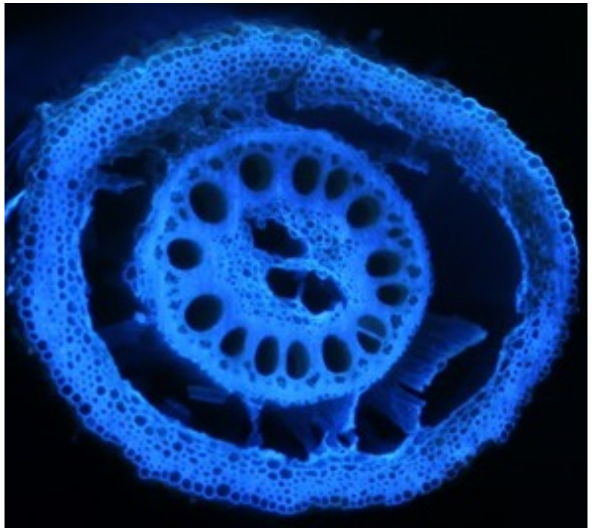

Supplemental figure 3. Cross section of root segment following tensilometry showing separation of the cortex from the stele highlighting that the cortex and stele have different biomechanical properties and that tissue structures separate during biomechanical testing.
